# Supplementary material for: Asynchronous magnetic resonance elastography: Shear wave speed reconstruction using noise correlation of incoherent waves
Source: Magn Reson Med. 2022 Oct 27;89(3):990–1001. doi: 10.1002/mrm.29502 (PMC9792433; doi:10.1002/mrm.29502)
Supplement: Supplementary file 1 — DATA S1 MATLAB code used to generate the numerical simulations of Figure 1 [file MRM-89-990-s002.zip › k-Wave/helpfiles/example_diff_binary_sensor_mask.html]

Using A Binary Sensor Mask (k-Wave)


# Using A Binary Sensor Mask

This example provides a simple demonstration of using k-Wave to model the diffusion of heat within a two-dimensional homogeneous medium with a constant source term (volume rate of heat deposition). It uses a binary source mask to extract the temperature time profile. It builds on the Heat Diffusion In A Homogeneous Medium and Constant Rate Of Heat Deposition examples.

- Open the file in the MATLAB Editor
- Run the file in MATLAB

## Contents

- Defining the sensor mask
- Turning the source on and off

## Defining the sensor mask

In the Heat Diffusion In A Homogeneous Medium and Constant Rate Of Heat Deposition examples, the sensor input is given as an empty array `[]` (i.e., it's not defined), and only the temperature after calling the `takeTimeStep` method is accessed. It is also possible to define a binary sensor mask in the same way as the other simulation functions in k-Wave (see the Using A Binary Sensor Mask Example). This allows the time history of the temperature field to be recorded. The current sensor data can be queried using the property `kdiff.sensor_data`, where `kdiff` is the name of the `kWaveDiffusion` object. The `sensor_data` is stored using MATLAB's standard column-wise linear matrix index ordering, indexed as `sensor_data(sensor_point_index, time_index)`. In this example, the sensor mask is defined with a single point in the centre of the grid.

```
% create binary sensor mask with a single sensor point in the 
% centre of the grid
sensor.mask = zeros(Nx, Ny);
sensor.mask(Nx/2, Ny/2) = 1;

% create kWaveDiffusion object
kdiff = kWaveDiffusion(kgrid, medium, source, sensor);
```

## Turning the source on and off

The main advantage of defining `kWaveDiffusion` as a class, rather than a conventional MATLAB function, is that the simulation properties can be modified, and the simulation continued. In particular, the volume rate of heat deposition can be changed to model a heat source which changes over time. In this example, a Gaussian shaped heat source is used to heat the tissue for 60 seconds. The heat source is then switched off, and the tissue is allowed to cool for 120 seconds.

```
% set source on time and off time
on_time  = 60;    % [s]
off_time = 120;   % [s]

% set time step size
dt = 0.1;

% take time steps
kdiff.takeTimeStep(round(on_time / dt), dt);

% turn off heat source
kdiff.Q = 0;

% take time steps
kdiff.takeTimeStep(round(off_time / dt), dt);
```

The temperature time curve at the sensor position in the centre of the grid is shown below. This shows a classic heating and cooling curve.
